# Supplementary material for: A set of multi-entry identification keys to African frugivorous flies (Diptera, Tephritidae)
Source: Zookeys. 2014 Jul 24;(428):97–108. doi: 10.3897/zookeys.428.7366 (PMC4143993; doi:10.3897/zookeys.428.7366)
Supplement: Supplementary material 6 — Key to Ceratitis [file zookeys-428-097-s006.zip › SF6_ZooKeys_key to Ceratitis/key/SF6_ZooKeys_key to Ceratitis/Media/Html/Ceratitis bremii.htm]

Ceratitis (Pardalaspis) bremii GuŽrin-MŽneville 


***Ceratitis*** **(*Pardalaspis*) *bremii* GuŽrin-MŽneville**

*Ceratitis* *bremii* GuŽrin-MŽneville, 1843: 199.

Body
length: 5.88 (5.0-7.0) mm; wing length: 6.25 (5.5-7.0) mm.

Male

Head.
Antennal segments yellow to yellow-orange. Third antennal segment twice as long
as second segment. Arista with basal third yellow, otherwise dark. Frons pale
to yellow ground colour, silvery over entire length; bare, no distinct hairs.
Ocellar triangle dark. Face pale; with median yellow band. At antennal socket
also with horizontal yellow band, sometimes both bands almost joining. Occiput
moderately swollen below, pale whitish. Chaetotaxy normal for subgenus.

Thorax.
Ground colour of mesonotum pale with orange tinge; no distinct spots, three
poorly defined darker stripes from anterior margin to dorsocentral setae,
darker area between dorsocentral setae and prescutellar acrostichal setae,
these markings not always distinct. Postpronotum same colour as centre of
mesonotum. Chaetotaxy normal for subgenus. Two anepisternal bristles. Mesonotum
with pale pilosity. Anepisternum completely pale pilose. Scutellum white
basally, otherwise yellow with apical markings black; basally without dark
spots, only slight darker coloration.

Legs.
Yellow; setation typical of subgenus, mainly pale especially on femora.
Posterior row on fore femur pale.

Wings.
Bands with yellow markings extensive. Banding, setation and venation normal for
subgenus. Marginal band continuous (in one specimen weakly interrupted); discal
band joined with marginal band; cross-vein r-m before middle of discal cell;
vein R1 ending beyond cross-vein r-m.

Abdomen.
Pale yellow, with weakly defined spots. Pattern of spots and setation normal
for subgenus.

Female

As
male except for the following characters: Frons yellower. Face with median band
indistinct. Marginal band partly interruped at vein R1. Oviscape shorter than abdominal terga 3-6.

(Description
after De Meyer, 1996)
